# ^11^C-Acetate PET Imaging in Patients with Multiple Sclerosis

**DOI:** 10.1371/journal.pone.0111598

**Published:** 2014-11-04

**Authors:** Kazushiro Takata, Hiroki Kato, Eku Shimosegawa, Tatsusada Okuno, Toru Koda, Tomoyuki Sugimoto, Hideki Mochizuki, Jun Hatazawa, Yuji Nakatsuji

**Affiliations:** 1 Department of Neurology, Osaka University Graduate School of Medicine, Suita, Osaka, Japan; 2 Department of Nuclear Medicine and Tracer Kinetics, Osaka University Graduate School of Medicine, Suita, Osaka, Japan; 3 Hirosaki University Graduate School of Science and Technology, Hirosaki, Aomori, Japan; 4 WPI-Immunology Frontier Research Center, Osaka University, Suita, Osaka, Japan; Research Inst. of Environmental Med., Nagoya Univ., Japan

## Abstract

**Background:**

Activation of glial cells is a cardinal feature in multiple sclerosis (MS) pathology, and acetate has been reported to be selectively uptaken by astrocytes in the CNS. The aim of this study was to investigate the efficacy of PET with ^11^C-acetate for MS diagnosis.

**Materials and Methods:**

Six patients with relapsing-remitting MS and 6 healthy volunteers (HV) were enrolled. The ^11^C-acetate brain uptake on PET was measured in patients with MS and HV. Volume-of-interest analysis of cerebral gray and white matter based on the segmentation technique for co-registered MRI and voxel-based statistical parametric analysis were performed. Correlation between ^11^C-acetate uptake and the lesion number in T1- and T2- weighted MR images were also assessed.

**Results:**

The standardized uptake value (SUV) of ^11^C-acetate was increased in both white and gray matter in MS patients compared to HV. Voxel-based statistical analysis revealed a significantly increased SUV relative to that in the bilateral thalami (SUVt) in a broad area of white matter, particularly in the subcortical white matter of MS patients. The numbers of T2 lesions and T1 black holes were significantly correlated with SUV of ^11^C-acetate in white and gray matter.

**Conclusions:**

The ^11^C-acetate uptake significantly increased in MS patients and correlated to the number of MRI lesions. These preliminary data suggest that ^11^C-acetate PET can be a useful clinical examination for MS patients.

## Introduction

Multiple sclerosis (MS) is an inflammatory demyelinating autoimmune disease of the CNS [1]. Although MRI is recognized as the most informative surrogate marker [2], the diagnostic value of MRI in MS remains insufficient [3]. Glial activation is a key feature in the neuroinflammatory MS pathology, and glial activation from the early phase of MS is suggested by a MRS study [4]. Microglial activation has also been shown in PET studies [5], [6]. However, astrocyte activation in MS has not been evaluated in vivo due to the lack of an appropriate radioligand, despite the astrocytosis observed from the early phase of disease and the important role potentially played by astrocytes [4], [7], [8].

Acetate is converted into fatty acids by the key enzyme acetyl-CoA synthase and metabolized in the citric acid cycle. ^11^C-acetate has been used as a tracer to evaluate cardiac oxidative metabolism [9] and later used as a PET biomarker in patients with renal cell carcinoma, hepatocellular carcinoma, prostate cancer, and multiple myeloma [10], [11], [12], [13]. In the CNS, ^11^C-acetate PET has proven useful for the diagnosis of astrocytoma [14] because acetate is preferentially absorbed into astrocytes by the monocarboxylate transporter (MCT) [15], [16]. Notably, the expression of MCT is increased in MS brains [17]. Therefore, we surmised that ^11^C-acetate PET could be a useful diagnostic tool in combination with MRI, and we investigated the utility of ^11^C-acetate PET for the diagnosis of MS and evaluated the astrocyte activity in the MS brain.

## Materials and Methods

### Subjects and clinical evaluation

Six patients with relapsing-remitting MS were evaluated. All patients were in the remission phase. Disability was assessed based on the Expanded Disability Status Scale (EDSS) [18]. Six healthy volunteers (HV) served as normal controls (Table 1). This study was approved by the Ethics Committee of Osaka University Hospital, and written informed consent was obtained from each participant.

**Table 1:** Patient data and demographics.

	sex	age	type	therapy	EDSS score	Disease duration (y)	GM SUVt	WM SUVt	WM/GM ratio
MS 1	F	47	RR	IFNβ, MTX	7	10.3	1.0494	0.9415	0.8972
MS 2	F	45	RR	IFNβ	2	6.8	1.1069	1.0529	0.9512
MS 3	F	48	RR	IFNβ	1	5.7	1.0282	0.8874	0.8631
MS 4	F	53	RR	IFNβ	2.5	7.4	1.0837	0.9434	0.8705
MS 5	F	34	RR	-	4	3.3	0.9633	0.8244	0.8558
MS 6	F	49	RR	-	1	1.4	1.0336	0.9044	0.8750
HV 1	F	54					0.9149	0.7320	0.8001
HV 2	F	61					0.9671	0.8121	0.8397
HV 3	F	41					0.9518	0.7789	0.8183
HV 4	F	67					0.9341	0.7946	0.8507
HV 5	F	62					0.9449	0.8021	0.8489
HV 6	F	63					0.9758	0.8096	0.8297

MS  =  multiple sclerosis, HV  =  healthy volunteer, RR  =  relapsing-remitting multiple sclerosis, IFNβ  =  interferon beta treatment, MTX  =  Mitoxantrone, EDSS  =  Expanded Disability Status Scale, SUV  =  standardized uptake value.

### MRI

MRI was performed using a GE SIGNA HDxt 3.0-T or a Phillips Achieva 3.0-T scanner. Three-dimensional (3D) structural MRI was performed for each subject using a T1-weighted spoiled gradient recalled (SPGR) sequence (axial plane; slice thickness, 0.90/0.95 mm; matrix size, 512×512; in-plane resolution, 0.47×0.47 mm; TR, 2.144 to 2.192/2.477 to 2.53 ms; TE, 6.908 to 7.108/6.000; flip angle, 18°/15°) and T2-weighted two-dimensional fast spin echo sequences (axial plane; FOV 250 mm; matrix size, 512×512; slice thickness, 5 mm; interslice gap, 1 to 1.5 mm; TE, 89/80 ms; TR, 4500/3000 ms).

### PET

PET was performed using a SET-3000 GCT/X scanner (Shimadzu Corp., Kyoto, Japan). ^11^C-acetate was synthesized by carbonation of Grignard reagent followed by acid hydrolysis. ^11^C-carbon dioxide reacted with methylmagnesium bromide followed by hydrolysis with hydrochloric acid to yield ^11^C acetic acid [19]. The radio chemical purity was greater than 98%. A total of 370 MBq of the tracer was administered intravenously, and a 20-min emission acquisition was initiated 20 min later. PET images were obtained in a 3-D mode. The images were reconstructed using a filtered-back projection method after 3D Gaussian smoothing with a 6-mm full width at half maximum (FWHM). Scatter correction was performed using a hybrid dual-energy window method combined with a convolution-subtraction method, and the true scatter-free component of the standard photopeak window was estimated sonographically. All PET images were reconstructed in 256×256×99 anisotropic voxels, with each voxel measuring 1×1×2.6 mm.

### Data analysis

#### Whole brain VOI analysis

All procedures were performed using a personal computer (DELL Precision T7400; DELL Inc., Round Rock, TX, USA) running on Microsoft Windows 7 (Microsoft Corp., Redmond, WA, USA). The 3D T1-weighted MRI scan was re-sliced in the native space of each subject using a 1.0×1.0×1.0 mm voxel size. The results were first categorized as GM, WM, and CSF, then spatially normalized using the unified model [20] of Statistical Parametric Mapping (SPM) 8 (Wellcome Department of Imaging Neuroscience: http://www.fil.ion.ucl.ac.uk/spm/) according to the optimized voxel-based morphometry (VBM) protocol [21]. This generated both spatial normalization matrices and inverse spatial normalization matrices. The resulting normalized GM map was transformed into native space using an inverse spatial normalization matrix. To generate VOI for GM and/or WM, binary mask images for the GM and/or WM were created using the segmented images in the native space of each subject. The binary mask image boundary was set at 35% of the maximum GM or WM concentration as described in previous studies [22], [23].

The ^11^C-acetate PET images were co-registered with the resliced 3D T1-weighted MRI using the SPM8 registration function based on the mutual information. The co-registration precision was inspected with the “Check Registration” tool in SPM8. Then, the co-registered PET images were spatially transformed using normalization and/or inverse normalization matrices identical to those generated in the previously described automatic segmentation process. The ^11^C -acetate uptake in the GM and WM VOI was analyzed using the binary masks within the native space.

To minimize contamination from the spill-in effect of adjacent brain segments, the spill-in-free VOIs of GM and WM were generated by the VOI erosion process. First, the binary masks were blurred by convolution using the point spread function of the PET scanner (presumably a simple isotropic Gaussian kernel with a FWHM of 8 mm). The spill-in-free gray matter mask 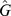 is expressed as follows:
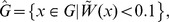
where 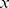 is a voxel, *G* is the gray matter binary mask, and 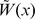 is the blurred image of the white matter binary mask (i.e. spill-in fraction from the white matter to the voxel 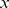). The spill-in-free white matter mask was also constructed as described above. Spill-in from CSF was assumed as zero. VOI analysis for ^11^C-acetate uptake using spill-in-free GM and WM masks was also performed (Figure S1).

The relative standardized uptake value (SUVt) served as the uptake indicator for analysis; the regional standardized uptake value (SUV) was divided by the mean SUV within the bilateral thalami of each subject. The Mann–Whitney *U* test was performed to determine significance of ^11^C -acetate SUVt differences between MS and HV. The significance level was designated at p<0.05.

#### Voxel-based statistical analysis

Voxel-based whole brain SUVt in the MS and HV groups was compared using Statistical Parametric Mapping (SPM) 8 (Wellcome Department of Imaging Neuroscience). The spatially normalized PET images were smoothed using a 12-mm FWHM isotropic Gaussian kernel, which conditions the residuals to conform more closely to the Gaussian random field model underlying the statistical adjustment of the p values. The SPM statistical model used voxel-by-voxel “two-sample T-test with covariates,” which designated age as a nuisance variable in order to detect voxels showing a significant age-adjusted SUVt difference between the MS and HV groups.

#### 11C-acetate uptake and the MR images correlation assessment

The T2 and T1 black hole lesions were independently recorded visually by three observers. T1 black holes were defined as visible hypointense regions on the T1- weighted images coincident with a high signal intense region on the T2-weighted images. Each MRI mask image was divided into its hemispheres to create the hemispheric VOIs. Pearson product moment correlation analyses were performed to assess the association between the number of MRI lesions, and the SUV of ^11^C-acetate was accessed from the hemispheric VOIs of the GM and WM. Statistical significance was designated at p<0.05.

### Statistical analysis

**Figure 1:**
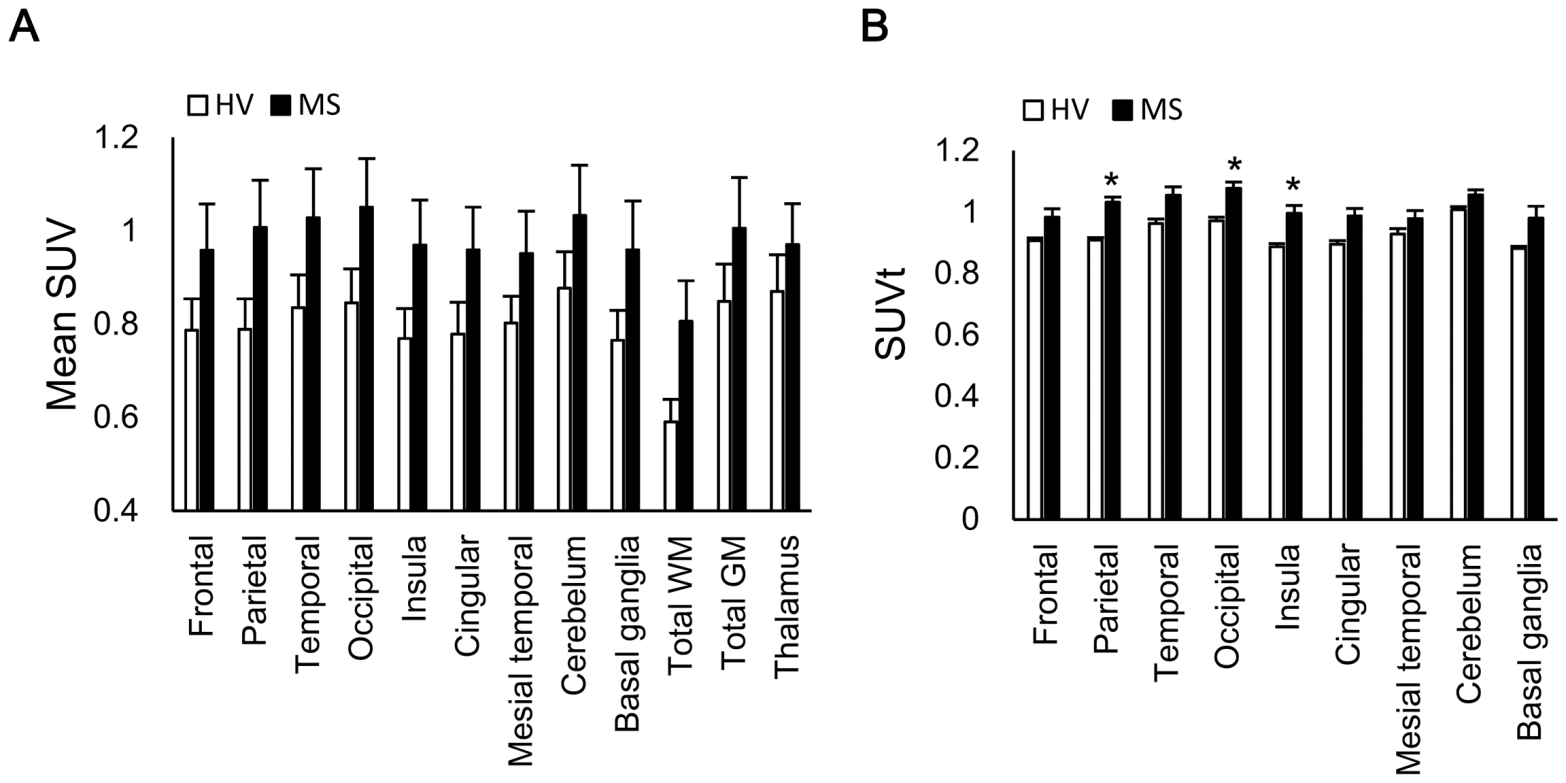
^11^C-acetate CNS biodistribution. (A)Mean standardized uptake value (SUV) of each lesion. (B) Relative SUV compared to that of the thalamus (SUVt). Data are expressed as the mean ± standard error of the mean (SEM) (n = 6). The Mann–Whitney *U* test showed a significant difference in the median between the HV and MS groups (*:p<0.0055 after Bonferroni correction). HV  =  healthy volunteers, MS  =  multiple sclerosis.

**Figure 2:**
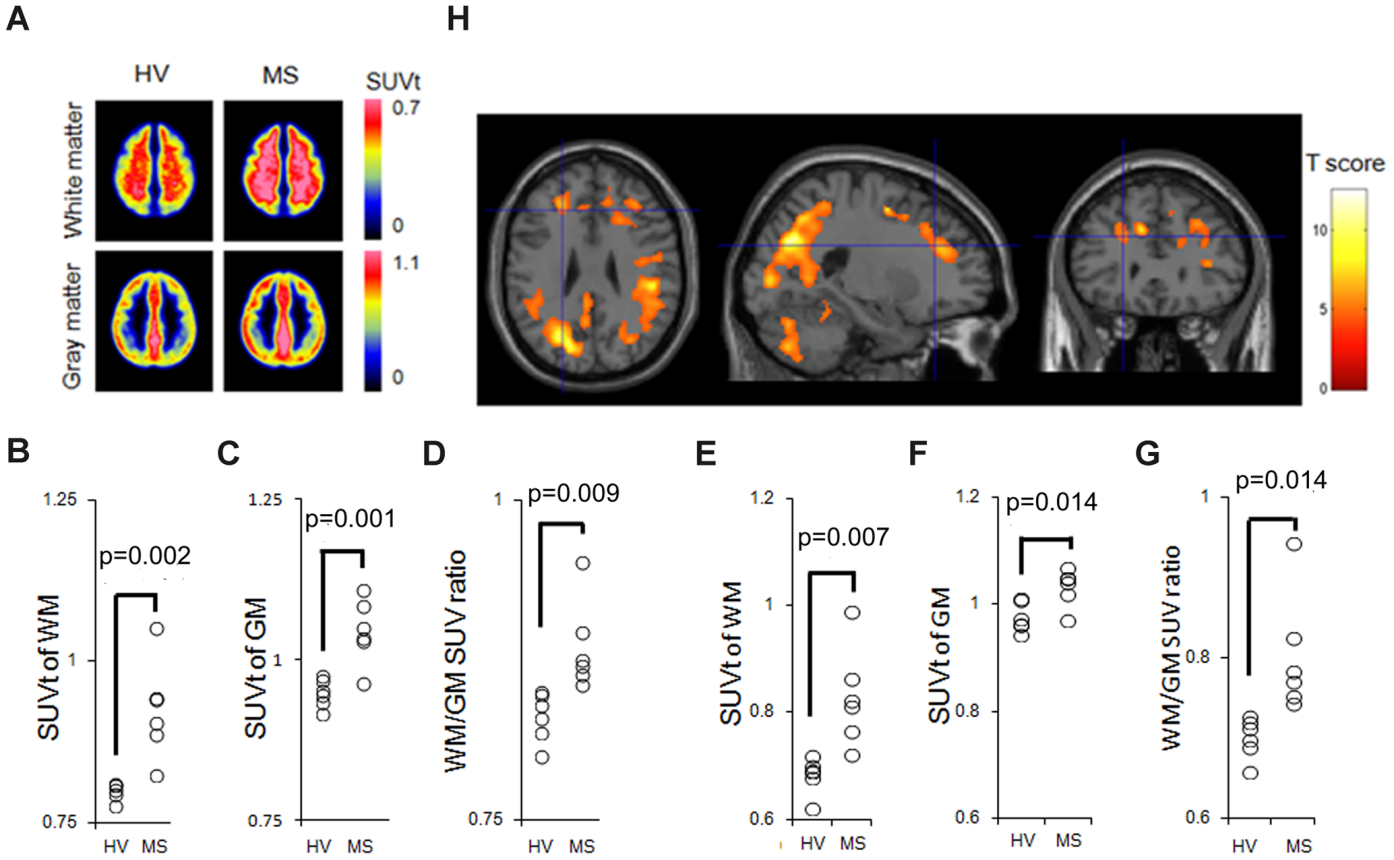
^11^C-acetate uptake distribution and quantification in MS patients. (A)Spatially normalized group mean images of ^11^C-acetate SUVt automatically segmented based on MRI. VOI analysis summarizing the mean SUVt in WM (B) and GM (C), and the WM/GM SUV ratio (D) in the HV and MS groups. The identical analysis performed using spill-in-free VOIs are also shown (E–G). The p-value was calculated using the analysis of covariance to adjust the variance of age. (H) The SPM analysis result is overlaid onto the T1-weighted brain MRI template. Colored voxels indicate T-scores representing significantly increased ^11^C-acetate uptake (SUVt) in patients with MS compared to HV patients. The spatially normalized PET images were smoothed for the analysis using a 12-mm FWHM isotropic Gaussian kernel. The significance thresholds are corrected for multiple comparisons at the cluster level with a p-value of 0.05 (family-wise error correction). SUV: standardized uptake value.

**Figure 3:**
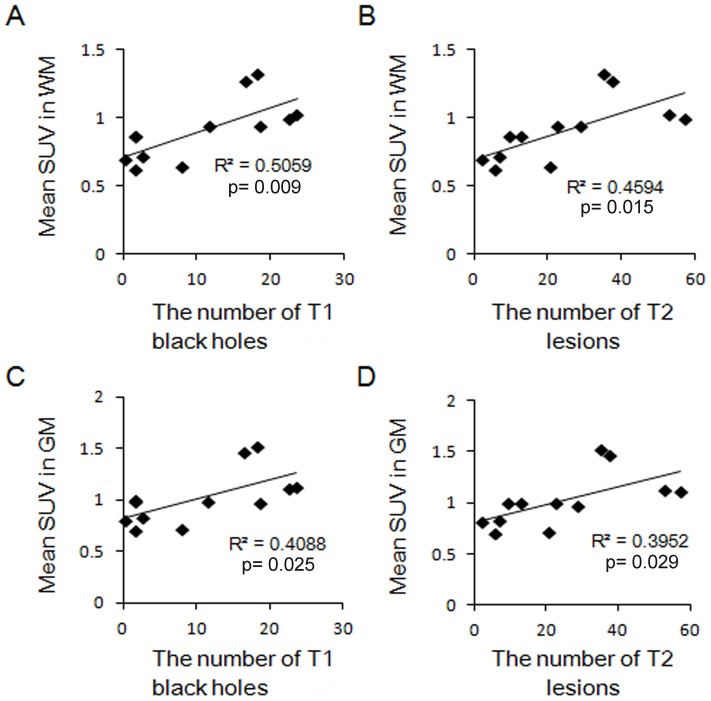
Correlation between ^11^C-acetate SUV and the number of MRI lesions in patients with MS. Correlation between ^11^C-acetate SUV in WM or GM and the number of T1 black holes (A, C) or T2 lesions (B, D) in each hemisphere of the six MS patients. SUV: standardized uptake value.

The data in Fig 1 and Table S1 were analyzed using the Mann–Whitney *U* test. ANCOVA was used to assess the differences between age-adjusted groups illustrated in Fig 2B–G, and Pearson product moment correlation analyses were performed for data in Fig 3, SPSS 14.J was used for statistical analysis.

## Results

VOI analysis of the ^11^C-acetate SUV revealed that the mean SUV was higher in the MS patients than in the HV in all regions assessed (Fig 1A). To evaluate the regional distribution of ^11^C-acetate uptake independent of physiologic variation in the whole brain, we calculated the relative uptake value (SUVt), which is the regional SUV divided by the mean SUV within the bilateral thalami of each participant (Fig 1B). The thalamus served as the reference region because it is rarely involved in MS pathology [24], and the SUV difference between the thalamus of HV and MS patients was the least among brain regions, as shown in Fig 1A. Each regional SUVt in the MS patients were increased particularly in the parietal, occipital, and insula regions.

Spatially normalized group mean images of ^11^C-acetate SUVt automatically segmented based on MRI showed increased uptake in both WM and GM in MS patients (Fig 2A). The SUVt of MS patients was significantly higher than that of HV in both WM (p = 0.002) and GM (p = 0.001). In addition, all six MS patients had a significantly higher WM/GM SUV ratio than the six HV (p = 0.009) (Fig 2B–D). This trend was consistently observed even after accommodating spill-in effect from adjacent brain segments (Fig 2E–G). Collectively, the ^11^C-acetate uptake significantly increased in both the WM and GM of MS patients, and this increase was more predominant in WM. The whole brain SPM analysis revealed a significant increase in SUVt of voxel cluster in MS patients compared to HV, primarily in the subcortical frontal, parietal, and occipital regions; no voxels showed a significantly lower SUVt in MS patients compared to HV (Fig 2H).

The voxel-based t-statistic for the WM tracts showed a significantly increased mean T-score, predominantly in the superior longitudinal fasciculus, posterior thalamic radiation, and sagittal stratum, with the highest local maximum T-score in the corpus callosum (Table S2).

We then assessed potential correlation between ^11^C-acetate SUV and MRI brain lesions. The mean SUV in WM was significantly correlated to the number of T1 black holes (R^2^ = 0.5059, p = 0.009) and T2 lesions (R^2^ = 0.4594, p = 0.015) (Fig 3A, B). The mean SUV in GM also correlated to the number of T1 black holes (R^2^ = 0.4088, p = 0.025) and T2 lesions (R^2^ = 0.3952, p = 0.029) (Fig 3C, D). The correlation to the EDSS score and disease duration did not reach statistical significance.

## Discussion

There have been few studies imaging astrocytes in vivo using ^11^C-acetate PET. In MS, astrocyte proliferation [25] and formation of scars composing a dense network of hypertrophic cells are characteristics of the MS histopathology [8]. An increased MCT expression in astrocytes within MS lesions was recently shown by immunohistochemical analysis [17], which suggest an increase in astrocyte metabolism. However, latent autoantibody-mediated astrocyte damage [26] supposedly decreases the metabolic activity, and therefore, the metabolic activity of astrocytes in MS brains remains undetermined. In this study, we observed a significantly increased brain uptake of the radioligand ^11^C-acetate in MS patients. Our study revealed for the first time that astrocytes are generally activated in MS brains based on the acetate metabolism.

Representative studies showed that a higher value in the kinetic parameter, which indicates the washout level of ^11^C-acetate, reflects the astrocyte reactivity in normal rats and healthy humans [27]. In MS, however, compared to HV, the pathologic changes in the severity of ^11^C-acetate accumulation may be much more prominent than the changes related to physiologic activation in healthy humans. Therefore, a slight increase in the washout speed may be inapparent in the PET SUV in MS. Furthermore, because the perfusion in the normal appearing white matter decreased in MS [28], the increase in ^11^C-acetate uptake by static PET may be underestimated due to a reduced CBF in MS.

The increased uptake was more pronounced in the WM, although a significant increase was observed in both the WM and GM. A significantly increased uptake was observed primarily within the subcortical WM on the voxel-based statistical analysis (Fig. 2H). On the voxel-based statistical analysis of the WM tracts, the distribution of the increased acetate uptake was similar to that in regions of axonal damage in DTI studies (Table S2). Recent voxel- and tract-based analyses in DTI studies revealed widespread damage to the subcortical WM, particularly in the sagittal stratum, corpus callosum, posterior thalamic radiation, and corona radiata [29]. These data suggested that the region-dependent increased acetate uptake was induced by the reactive astrocyte coexisting with heterogeneously dispersed MS lesions detected in DTI studies (Fig. 2H and Table S2). Although inflammatory WM demyelination detected by conventional MRI is a cardinal feature of MS, pathologic changes exist even in normal appearing WM and GM [30]. Astrocyte pathology precedes demyelination in an animal model [31]; astrocyte hypertrophy occurs at the leading edge of acute MS lesions, followed later by astrocytic scarring [8]. Thus, the altered astrocyte activation is presumably involved in MS pathophysiology [4], [7], [32]. Correlation between the radial diffusivity quantified by DTI and T1 black hole formation are recognized markers of axonal loss and tissue destruction [33], [34]. In the present study, the strongest correlation was detected between the mean SUV in WM and the T1 black hole number, suggesting that the mean SUV may correlate with axonal damage. The mean SUV in GM also increased and correlated with the number of MRI lesions, suggesting cortical astrocyte involvement in MS pathology. Cortical involvement and subsequent cognitive decline occur in approximately half of MS patients [35]. However, little information exists on the pathophysiologic involvement of cortical astrocytes [36]. Normally, astrocytes supply lactate to neurons for oxidation [37], and metabolic dysfunction of neurons and glial cell activation likely occurs in the MS brain [25]. Moreover, astrocytes are associated with preclinical axonal damage in an animal model of MS [38]. These results suggest that the increased ^11^C-acetate uptake within GM may reflect astrocyte-associated cortical damage in MS.

The present study has a few limitations. First, ^11^C-acetate uptake in MS plaques was not assessed separately because most plaques were so small that a partial volume effect caused by the relatively low resolution of PET was inevitable. Second, the analysis was performed on static PET data instead of kinetic parameters. In the present study, the data acquired between 20 to 40 min after tracer administration were summed to build static uptake images because the time activity curve stabilized after 20 min (data not shown). Regional uptake distribution may be contaminated by the dispersion of radioactive metabolites. However, in our study, 1-^11^C-acetate was used, and its dispersion of labeled metabolites was the smallest among the various types of acetate tracers [39], [40]. In addition, because almost all the tracer was first absorbed through MCT-1 expressed within astrocytes according to their reactivity, the summed radioactivity is thought to reflect the first uptake of ^11^C- acetate and its subsequent metabolism by reactive astrocytes. Finally, because the mean age was higher in the control group than in the MS group, we used the ANCOVA to assess the differences among the age-adjusted SUVt. Although the mean age of MS patients was generally lower than that of the healthy volunteers, age did not significantly affect the increased uptake of ^11^C-acetate in MS patients.

## Conclusions

The present study suggests that the pathologic white matter changes in patients with MS can be detected by non-invasive static ^11^C-acetate PET, which may be an effective MS diagnostic tool. Development of clinically applicable monocarbonic acid tracers labeled with longer half-life radioactive nuclides are needed, as are further studies enrolling more participants, including those in the early and relapse phases.

## Supporting Information

Figure S1 **Binary mask imaging parameters for VOI analysis.** The scheme of VOI analysis is described. A: ^11^C-acetate PET, B: 3D MRI, C: Co-registration, D: Spatial normalization to the MNI space, E/F: Segmented GM/WM map in the MNI space, G/H: GM/WM binarized mask in the original space of the subject, I/J: Eroded version of G/H for spill-in-free VOI analysis, K–N: GM/WM masks overlaid onto PET in the original space of the subject. MNI: montreal neurological institute. f: Transformation matrix for spatial normalization, f^−1^: Inverse of the transformation.
(TIF)

Table S1 **Relative ^11^C-acetate biodistribution in the CNS.** The mean SUVt of each lesion in the CNS was analyzed and for group comparison between HV and MS patients, the Mann–Whitney *U* test was performed.
(DOC)

Table S2 **Regional T-scores from voxel-based statistical comparison in WM.** Voxel-based statistical comparison in white matter tracts was performed. The positive T-scores indicate an increased ^11^C-acetate uptake in the MS patients compared to the HV.
(DOC)

Text S1 **Supplementary methods.** Methods for “Voxel-based statistical analysis for WM tracts” are described with references.
(DOC)
