# Supplementary figures and images for: 11C-Acetate PET Imaging in Patients with Multiple Sclerosis
Source: PLoS One. 2014 Nov 4;9(11):e111598. doi: 10.1371/journal.pone.0111598 (PMC4219725; doi:10.1371/journal.pone.0111598)

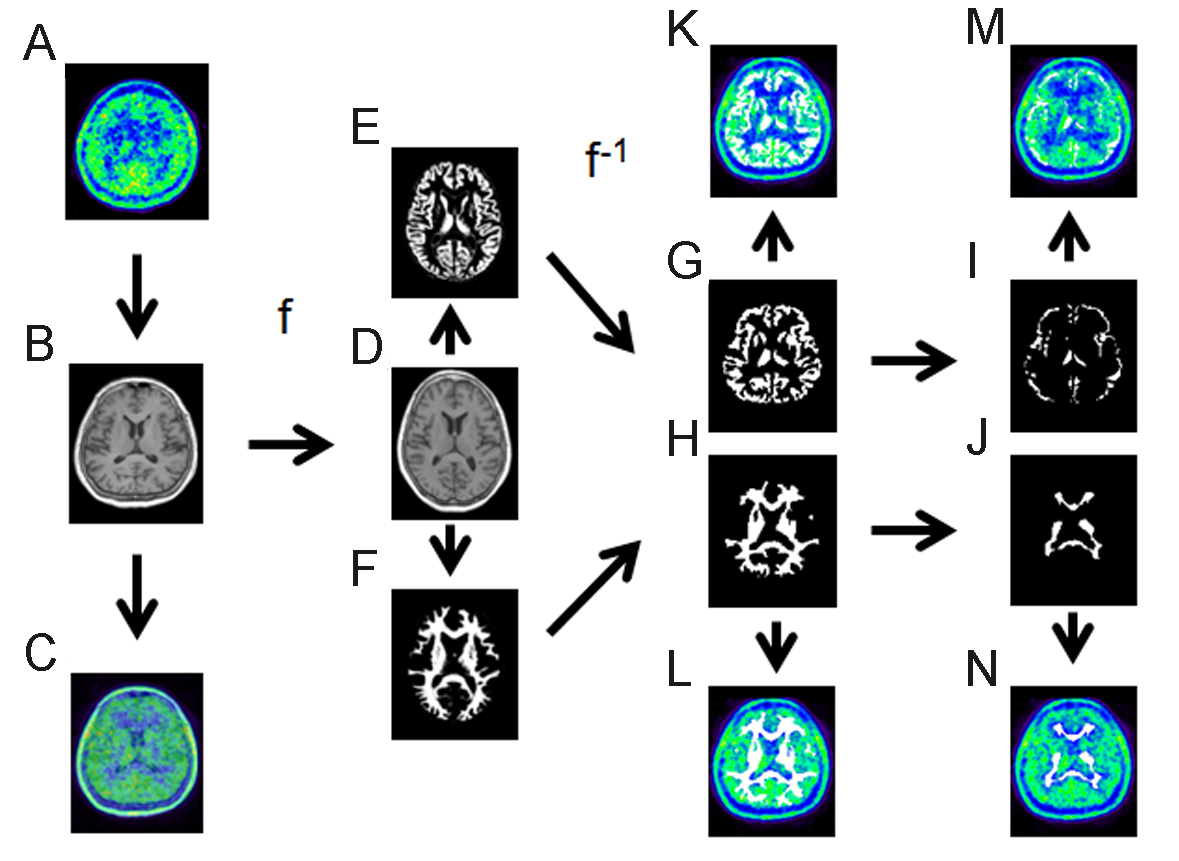

Supplement: Figure S1 — Binary mask imaging parameters for VOI analysis. The scheme of VOI analysis is described. A: 11C-acetate PET, B: 3D MRI, C: Co-registration, D: Spatial normalization to the MNI space, E/F: Segmented GM/WM map in the MNI space, G/H: GM/WM binarized mask in the original space of the subject, I/J: Eroded version of G/H for spill-in-free VOI analysis, K–N: GM/WM masks overlaid onto PET in the original space of the subject. MNI: montreal neurological institute. f: Transformation matrix for spatial normalization, f−1: Inverse of the transformation. (TIF) [file pone.0111598.s001.tif]
