# Supplementary material for: 11C-Acetate PET Imaging in Patients with Multiple Sclerosis
Source: PLoS One. 2014 Nov 4;9(11):e111598. doi: 10.1371/journal.pone.0111598 (PMC4219725; doi:10.1371/journal.pone.0111598)
Supplement: Table S1 — Relative 11C-acetate biodistribution in the CNS. The mean SUVt of each lesion in the CNS was analyzed and for group comparison between HV and MS patients, the Mann–Whitney U test was performed. (DOC) [file pone.0111598.s002.doc]

**Table S1. Relative ^11^C-acetate biodistribution in the CNS.**

|  | HV | SEM | MS | SEM | p value* |
| --- | --- | --- | --- | --- | --- |
| Frontal | 0.9070 | 0.0082 | 0.9838 | 0.0265 | 0.0931 |
| Parietal | 0.9086 | 0.0087 | 1.0324 | 0.0174 | 0.0022 |
| Temporal | 0.9625 | 0.0154 | 1.0545 | 0.0280 | 0.0152 |
| Occipital | 0.9725 | 0.0103 | 1.0783 | 0.0191 | 0.0022 |
| Insula | 0.8866 | 0.0101 | 0.9955 | 0.0258 | 0.0043 |
| Cingular | 0.8960 | 0.0114 | 0.9881 | 0.0239 | 0.0087 |
| Mesial temporal | 0.9286 | 0.0171 | 0.9792 | 0.0258 | 0.1797 |
| Cerebellum | 1.0071 | 0.0112 | 1.0561 | 0.0164 | 0.0260 |
| Basal ganglia | 0.8812 | 0.0068 | 0.9807 | 0.0388 | 0.0411 |

Data showed mean SUVt of each lesion. *: For group comparison, the Mann–Whitney *U* test was used.

SEM = standard error of the mean, HV = healthy volunteer, MS = multiple sclerosis.
