# Supplementary material for: 11C-Acetate PET Imaging in Patients with Multiple Sclerosis
Source: PLoS One. 2014 Nov 4;9(11):e111598. doi: 10.1371/journal.pone.0111598 (PMC4219725; doi:10.1371/journal.pone.0111598)
Supplement: Table S2 — Regional T-scores from voxel-based statistical comparison in WM. Voxel-based statistical comparison in white matter tracts was performed. The positive T-scores indicate an increased 11C-acetate uptake in the MS patients compared to the HV. (DOC) [file pone.0111598.s003.doc]

**Table S2. Regional T-scores from voxel-based statistical comparison in WM.**

| WM tracts | T mean | T max† |
| --- | --- | --- |
| Superior longitudinal fasciculus***** | 3.553 | 6.954 |
| External capsule | 2.642 | 5.018 |
| Cingulum | 2.483 | 6.957 |
| Sagittal stratum***** | 2.936 | 5.338 |
| Fornix | 2.147 | 3.453 |
| Superior fronto-occipital fasciculus | 1.948 | 3.138 |
| Corpus callosum | 2.021 | 8.521 |
| Uncinate fasciculus | 1.691 | 3.63 |
| Corona radiata | 2.694 | 5.482 |
| Internal capsule | 2.612 | 5.588 |
| Posterior thalamic radiation***** | 3.401 | 6.27 |
| Cerebellar peduncle | 1.561 | 5.219 |
| Cerebral peduncle | 1.018 | 2.676 |
| Corticospinal tract | -1.055 | -3.634 |
| Medial lemniscus | -1.192 | -3.271 |

The positive T-scores indicate an increased ^11^C-acetate uptake in the MS patients compared to the HV. *: The region containing significant voxels (corrected for multiple comparisons at the cluster level at a p-value of 0.05 [family-wise error correction]). †: Maximum absolute value of the T-score.
