# Supplementary material for: 11C-Acetate PET Imaging in Patients with Multiple Sclerosis
Source: PLoS One. 2014 Nov 4;9(11):e111598. doi: 10.1371/journal.pone.0111598 (PMC4219725; doi:10.1371/journal.pone.0111598)
Supplement: Text S1 — Supplementary methods. Methods for “Voxel-based statistical analysis for WM tracts” are described with references. (DOC) [file pone.0111598.s004.doc]

**Text S1. Supplementary methods**

*Voxel-based statistical analysis for WM tracts*

Disease-related change in ^11^C-acetate SUVt of the WM tracts was accessed by limiting the voxel-based targeted statistical inference to the WM tracts. The two-sample T-test was performed with the “explicit masking” option in the “estimation” process using WM tract atlases in stereotaxic coordinates based on diffusion tensor imaging “JHU-ICBM-DTI-81 WM atlas” [1].

For WM tracts, an automatic atlas-based VOI analysis for the regional T-score was performed using on-site software as described previously [2]. Briefly, the T-score maps generated from the SPM analysis were accessed using this software tool, by automatically calculating the mean and maximum T-score in each WM tract within the maps in the Montreal Neurological Institute (MNI) space. The positive T-score served as an indicator of increased SUVt in the MS patients compared to HV patients.

**References**

1. Mori S, Oishi K, Jiang H, Jiang L, Li X, et al. (2008) Stereotaxic white matter atlas based on diffusion tensor imaging in an ICBM template. Neuroimage 40: 570-582.

2. Kato H, Shimosegawa E, Isohashi K, Kimura N, Kazui H, et al. (2012) Distribution of cortical benzodiazepine receptor binding in right-handed healthy humans: a voxel-based statistical analysis of iodine 123 iomazenil SPECT with partial volume correction. AJNR Am J Neuroradiol 33: 1458-1463.
